# Systematic Review of Multi-Omics Approaches to Investigate Toxicological Effects in Macrophages

**DOI:** 10.3390/ijms21249371

**Published:** 2020-12-09

**Authors:** Isabel Karkossa, Stefanie Raps, Martin von Bergen, Kristin Schubert

**Affiliations:** 1 Department of Molecular Systems Biology, Helmholtz-Centre for Environmental Research—UFZ, 04318 Leipzig, Germany; isabel.karkossa@ufz.de (I.K.); stefanie.raps@ufz.de (S.R.); martin.vonbergen@ufz.de (M.v.B.); 2 Institute of Biochemistry, Leipzig University, 04103 Leipzig, Germany

**Keywords:** multi-omics, macrophages, toxicology, xenobiotics

## Abstract

Insights into the modes of action (MoAs) of xenobiotics are of utmost importance for the definition of adverse outcome pathways (AOPs), which are essential for a mechanism-based risk assessment. A well-established strategy to reveal MoAs of xenobiotics is the use of omics. However, often an even more comprehensive approach is needed, which can be achieved using multi-omics. Since the immune system plays a central role in the defense against foreign substances and pathogens, with the innate immune system building a first barrier, we systematically reviewed multi-omics studies investigating the effects of xenobiotics on macrophages. Surprisingly, only nine publications were identified, combining proteomics with transcriptomics or metabolomics. We summarized pathways and single proteins, transcripts, or metabolites, which were described to be affected upon treatment with xenobiotics in the reviewed studies, thus revealing a broad range of effects. In summary, we show that macrophages are a relevant model system to investigate the toxicological effects induced by xenobiotics. Furthermore, the multi-omics approaches led to a more comprehensive overview compared to only one omics layer with slight advantages for combinations that complement each other directly, e.g., proteome and metabolome.

## 1. Introduction

One step towards a facilitated risk assessment of xenobiotics is to identify approaches, which allow to decipher modes of action (MoAs). Generally, xenobiotics can be a molecular initiating event (MIE), followed by causally related intermediate events, also called key events (KEs), resulting in an adverse outcome (AO), which is referred to as adverse outcome pathway (AOP) [1]. Unravelling AOPs is relevant for risk assessment and the regulation of xenobiotics since it allows to understand undesirable effects better [1,2].

### 1.1. Immune Cells Are a Relevant Model System to Investigate Toxicity

The immune system is an interactive and complex network of lymphoid organs, immune cells, and humoral factors. The cells of the immune system can recognize foreign substances, invading pathogens, and allergens at an early stage to protect their hosts from infections. The adaptive immune defense is formed by T- and B- lymphocytes, while granulocytes, mast cells, monocytes, macrophages, and dendritic cells (DCs) are part of the innate immune system [3,4].

It is well-known that the exposure to xenobiotics can be perceived as a stress factor by cells of the immune system and can cause inflammation [5,6]. Inflammation is a biologically complex and adaptable process of the human body to eliminate foreign substances or pathogens. Thus, xenobiotics can lead to acute or chronic inflammatory reactions, which may trigger several diseases, even cancer [7,8]. In general, the immunoregulatory effects of a xenobiotic are considered immunotoxicity, which is accompanied by suppressive or enhancing effects onto the immune system [9,10,11]. For example, effects of chromium on cells of the immune system have been described by Shrivastava et al., 2002, where immunoregulatory effects of chromium ions on lymphocytes, macrophages, and the immune response (e.g., cytokine production and cell death) were observed [12]. Furthermore, Forawi et al., 2004 described immunomodulatory mechanisms of TCDD- and DDT- induced toxicity [13]. Mother–child cohort studies have shown that exposure to xenobiotics in the prenatal and postnatal phase can influence the maturation of the immune system and thus affect the risk of diseases in children. For example, an increased phthalate exposure of the mother during pregnancy and breastfeeding has been linked to a higher risk of allergies in children [14]. Also, lower numbers of regulatory T cells have been described after prenatal phthalate exposure [15]. Additionally, the accumulation of persistent organic pollutants in adipose tissue has been shown to correlate with markers of glycemia, insulin sensitivity, and notably also inflammation, indicating that certain xenobiotics affect systemic processes. Thereby, differences between lean and obese individuals have been observed, highlighting the role in the state of health [16].

Importantly, each xenobiotic can have distinct immunotoxicological effects and consequently, an individual underlying MoA, which also results in different AOPs. One KE within the AOP framework has been described to be inflammation, which is now considered a link between a series of AOPs that previously had no shared KEs [17], thus underlining the relevance of the immune system when assessing the toxicity of xenobiotics. The definition of immune-mediated AOPs is currently emerging. For example, Selvaraj et al., 2020 described an AOP for immune-mediated and allergic hepatitis, where an immune-mediated inflammatory cascade in mouse experiments led to hepatitis after treatment with diclofenac [18]. Also, AOPs for nanomaterials with hepatitis and liver fibrosis as AOs and inflammatory processes as KEs are currently under development [19]. Thus, xenobiotics have already been shown to induce AOs on various cells of the immune system, highlighting that immune cells are relevant for the investigation of toxicological effects of xenobiotics.

### 1.2. The Innate Immune System Is the First Barrier against Foreign Substances

Pathogens, xenobiotics or in general foreign substances that enter the organism are recognized by the immune system, which can differentiate between self and foreign, thus protecting the host from an impending infection. If physical or chemical barriers are damaged due to exposure, a local (e.g., in contact-related hypersensitivity) or systemic (e.g., in autoimmune diseases) infection can occur, which depends on many factors (e.g., type of xenobiotic, duration of exposure, distribution in the organism). Importantly, the first protective instance against invaded pathogens or xenobiotics is the innate immune system [3,4,20], which reacts immediately (but rather unspecific) and can recognize damage-associated molecular patterns (DAMPs) or pathogen-associated molecular patterns (PAMPs), in turn activating pattern recognition receptors [21,22]. A typical example is the activation of toll-like receptor 4 (TLR4) by lipopolysaccharide (LPS) [23] in innate mononuclear phagocyte-like macrophages. Furthermore, the activation of human TLR4 by the contact allergen nickel has been described before [24]. Antigen-presenting cells, like DCs or macrophages, phagocytose foreign substances and present fragments (antigens) on their surface to activate the adaptive immune system. T helper cells can recognize these antigens and trigger cell-mediated immune responses or activate B cells, which mature into plasma cells and produce antibodies [25,26]. Regulatory or cytotoxic T cells are also subsets of T lymphocytes. In recent years, mucosal-associated invariant T cells (MAIT cells), a population of innate-like T lymphocytes, have come into focus because of their role in several diseases [27,28], e.g., liver diseases [29].

Since macrophages are part of the innate immune system and thus one of the first barriers against xenobiotics entering the body, we focused on the effects of xenobiotics on macrophages. Macrophages are a heterogeneous population of cells with two macrophage phenotypes being distinguished, M1 and M2 [30,31,32]. Upon activation, M1 macrophages promote the release of pro-inflammatory mediators, whereby more leukocytes are recruited and activated, thus initiating inflammation. Subsequently, circulating monocytes can migrate to the site of inflammation and differentiate into macrophages. On the other hand, the anti-inflammatory M2 macrophages play a role in maintaining tissue homeostasis as well as resolving inflammation. Consequently, M2 macrophages are involved in tissue repair [31]. Tissue-resident macrophages, which arise during embryogenesis and can be referred to as specialized tissue macrophages, also play a decisive role in the resolution of inflammation and wound healing [33]. Importantly, persistent inflammation can lead to tissue dysfunction and can trigger various AOs in the form of diseases, e.g., atherosclerosis, asthma, obesity, and cancer [34].

Notably, M1 and M2 macrophages show significant differences in their immunometabolism. In M2 macrophages, the tricarboxylic acid cycle (TCA) is intact, and fatty acid oxidation, as well as the production of anti-inflammatory products, are increased. In contrast, M1 macrophages exhibit elevated glycolysis, pentose phosphate pathway, and fatty acid synthesis, while the TCA is diminished. Thus, phagocytic and microbicidal functions, as well as the production of pro-inflammatory lipid intermediates, cytokines, and the production of reactive oxygen species (ROS) and reactive nitrogen intermediates, are raised in M1 compared to M2 macrophages [35,36].

Key regulators of the release of pro-inflammatory cytokines in macrophages are inflammasomes, of which for instance, the NLR family pyrin domain containing 3 (NLRP3) inflammasome can be activated by PAMPs and DAMPs [37]. Importantly, differences in the induction of the NLRP3 inflammasome have been described for M1 and M2 macrophages [38]. Furthermore, it has been observed that the NLRP3 inflammasome can be affected by xenobiotics like cadmium [39] and statins in endothelial cells [40]. Thereby, the statins acted via the pregnane X receptor (PXR), which is related to the detoxification of xenobiotics and has been shown to affect the induction of the NLRP3 inflammasome in endothelial cells [41] and macrophages [42]. Additionally, the NLRP3 inflammasome can be activated in response to ROS [43] as described for silica and asbestos [44]. In contrast, the activation of the aryl hydrocarbon receptor (AhR), which can bind to the xenobiotic response element (XRE) in the NLRP3 promoter has been shown to inhibit the NLRP3 transcription in macrophages, thus being a negative regulator of the NLRP3 inflammasome activity [45]. 

Remarkably, an often-used model system mimicking macrophages functions in vitro is the human leukemia monocytic cell line THP-1 [46,47], but U937, HL-60, ML-2, or Mono Mac 6 cells are also well-established model systems. All have in common that they arise from immature cells of the monocytic differentiation line. While for example, U937 cells originate from the tissue, THP-1 cells were isolated from the blood, thus being less mature than tissue-derived monocytes [47]. However, THP-1 cells have been often used in toxicology to study nanomaterials (NMs) [48,49,50,51] and other xenobiotics [52,53]. Besides human cell lines, rodent cell lines are a popular model system to answer macrophage-related immunotoxicological questions [54,55], because they allow the comparison to in vivo studies that are usually conducted in mice or rats. One example is the male macrophage cell line RAW 264.7 [56,57], in which Park et al., 2011 could demonstrate cell membrane damage, decreased metabolic activity, ROS and production of various cytokines due to treatment with silica NMs [58].

### 1.3. Omics Approaches Are Advantageous to Decipher the MoAs of Xenobiotics

So far, mainly conventional toxicological methods are used to assess the toxicity of xenobiotics in macrophages. For example, Hubbard et al., 1999 provided a basic overview of methods that can be used to evaluate the effects of xenobiotics on macrophages, including methods assessing, the formation of ROS or NO, cytokine activity, or cytotoxicity [54]. Furthermore, Lee et al., 2011, who studied mouse macrophages, examined the effects of silica NMs, focusing on single parameters reflecting cytotoxicity, oxidative stress, apoptosis, and inflammation [59]. Also, Wiemann et al., 2016 suggested single parameters like lactate dehydrogenase and glucuronidase as membrane damage markers, tumor necrosis factor as inflammation marker, and H_2_O_2_ as oxidative stress marker to be suitable to distinguish active from passive NMs [55].

In contrast to those conventional toxicological methods, which usually investigate toxicological endpoints, omics approaches allow a comprehensive investigation of biological pathways. Thus, a systematic understanding of the underlying MoAs upon exposure to xenobiotics can be revealed [60,61]. Furthermore, statements on risk assessment, for example in the form of the classification of substances is possible [62] as well as the development of AOPs, which might facilitate future risk assessment [63,64]. From the list of available omics approaches, we here focus on transcriptomics, proteomics, and metabolomics for the investigation of toxicological effects in macrophages in response to xenobiotic exposure.

### 1.4. Different Omics Approaches Exhibit Diverse Insights into MoAs That Can Complement Each Other

#### 1.4.1. Transcriptomics

Transcriptomics are used to detect changes in ribonucleic acid (RNA) levels within the cell and thus provide information at the regulatory level. Thereby, it can be distinguished between protein-coding RNA (mRNA) and non-coding RNA (ncRNA), which allow the identification of affected pathways [65]. Also, the detection of other RNAs, like circular RNA [66] could become a new option for transcriptional analysis. There are several available approaches for transcriptional analysis. On the one hand, microarrays are often used as demonstrated for a variety of xenobiotics that were analyzed concerning hepatotoxicity and pulmonary toxicity [67]. Microarrays are based on hybridization of DNA, whereby mRNA is first obtained from biological samples for the synthesis of cDNA via reverse transcription. The subsequent detection of cDNAs is based on probes and hybridization. For example, the adhesion of monocytic MM6 cells after exposure to aqueous cigarette smoke has been examined using microarrays [68] as well as research on zinc oxide NMs in THP-1 cells [69]. Importantly, transcriptomics applying microarrays is a targeted method, where only transcripts can be identified that were selected and thus known before. In contrast, transcriptome sequencing (RNA-seq) is an untargeted approach, which is based on the reverse transcription into cDNA in a first step as well and which allows to identify different RNAs from one sample [70,71]. Also, RNA-seq has been already used for the detection of changes on the transcriptome level for toxicological questions [72,73]. Demsey et al., 2017 describes long non-coding RNAs as a key regulator of toxicological reactions that can be triggered by various xenobiotics and connects these with different clinical diseases [74]. Furthermore, RNA-seq has already been used to investigate changes during the differentiation of human monocytes into macrophages [75]. Even the influence of silica NMs on RAW 264.7 cells has been described with this method before [76].

#### 1.4.2. Proteomics

With proteomics, the entirety of all proteins in a biological system can be recorded with the possibility to detect post-translational modifications as well, which was not focused on in the present study. In proteomics, the analysis and quantification of expressed proteins are carried out using mass spectrometry (MS)-based methods. For in vitro investigations of metals [77,78,79] or NMs [80,81,82,83] in human monocyte cell lines or macrophages (human or murine), the rather obsolete method of 2D-gel-based proteomics is commonly used. With this method, the proteins are separated and quantified using 2D-gel electrophoresis based on mass and isoelectric point, and chosen spots are identified with tandem MS (MS/MS) [84]. Notably, the state-of-the-art in proteomics are untargeted analyses, also called shotgun analyses, where the global proteome is investigated. Usually, liquid chromatography (LC)-MS/MS is used for the identification and quantification of a large part of the proteome in shotgun proteomics. For the quantification, two approaches can be distinguished, namely label-free and label-based quantification. For both methods, the identification of peptides is performed based on the MS2 level, where fragment ions of the separated peptides are recorded, which give information on the amino acid sequence of the original peptide. The major difference between the methods lies in the quantification. In label-free quantification (LFQ) approaches, the quantification is performed based on the MS1 level and thus, the elution profiles of the peptides [85,86]. This method is regularly applied for the elucidation of pathological pathways and biomarkers and thus for the identification of drug targets in relation to various diseases in clinical proteomics [87]. Also, the label-based stable isotope labeling by amino acids in cell culture (SILAC), where different isotopes are introduced into the cell culture, thus leading to isotopically labeled proteins and peptides [88], uses the MS1 level for quantification. Hwang et al., 2016 used this method to study the toxicological effects of 2,3,7,8-tetrachlorodibenzo-p-dioxin (TCDD) on the AhR in mouse cell lines identifying the mitochondrial proteome [89]. In contrast, isobaric tags like tandem mass tags (TMT) can be used to label peptides after enzymatic cleavage of proteins for shotgun proteomics. These tags can be detected in MS2 spectra and used for the quantification of the peptides in the respective samples [90]. Various glycosylation sites in lysosomes could be identified in RAW 264.7 macrophages as a response to different bacteria and viruses by TMT labeling. Furthermore, the TMT approach has been used to investigate the effects of the antifungal drug ketoconazole in a co-culture model of THP-1 cells and hepatocytes as well as in the single cultures [91]. A decisive advantage using TMT is multiplexing, whereby proteins from up to ten samples (nowadays even up to sixteen samples) can be combined and quantified relative to each other [90,92]. Thus, high reproducibility and minimal measuring time are guaranteed. With SILAC, only three samples can be combined [88], whereas, with LFQ, only one sample can be measured at a time [85,86]. However, targeted analysis using multiple reaction monitoring (MRM) or parallel reaction monitoring (PRM) is also possible, whereby a list of isotope-labeled selected peptides is isolated explicitly from the MS1 spectra and then fragmented. Thus, a less comprehensive overview of affected proteins is revealed, but importantly, these methods allow the reproducible quantification of low-abundant proteins from complex mixtures [93,94,95,96,97]. Consequently, MRM is used for quantitative biomarker development, not only in proteomics but also in metabolomics [98].

#### 1.4.3. Metabolomics

The metabolome describes the metabolites in a biological system, and the analysis of all these chemically heterogeneous small biological molecules is summarized under the term metabolomics [99]. Targeted and untargeted methods can be used for metabolomics [100], and both approaches have already been used for toxicity-related investigations [101]. The targeted procedure can be used to answer precise scientific questions, and the samples can be quantified either relative to each other or absolutely with the help of stable isotope-labeled reference standards, which can be detected by MS/MS [98,101]. An often applied tool in targeted metabolomics is the AbsoluteIDQ p180 kit (Biocrates), which allows the standardized identification and quantification of up to 188 metabolites and has been applied to toxicological questions before [102,103,104]. Recently, the MxP^®^ Quant 500 kit (Biocrates) has been introduced and used by the first groups [105,106], which covers up to 630 metabolites, thus allowing a more comprehensive profiling of metabolomic changes. In contrast to targeted methods, the untargeted approach is usually performed label-free and aims to quantify as many endogenous small molecules as possible to get insights into MoAs [107]. Due to the complexity of the metabolome and the high variability in the properties of metabolites, no single approach can be used to detect all metabolites in one biological sample [108,109]. Thus, compared to proteomics, where usually LC is used to separate the peptides, also gas chromatography (GC) can be advantageous for metabolite separation, depending on the targets. Also, nuclear magnetic resonance (NMR) is often used as an analysis method in metabolomics [100,101]. Both targeted and untargeted metabolomics have been applied to investigate effects on immune cells [110,111,112,113], macrophages [114,115,116,117], or toxicological questions in general [118,119,120] before. However, investigations of xenobiotic stimuli on macrophages using metabolomics are still very rare [121,122] compared to transcriptomics and proteomics.

### 1.5. Multi-Omics Approaches Allow a Comprehensive Overview of Toxicological Effects in Macrophages

While single omics approaches already allow the in-depth analysis of xenobiotic-induced effects, a combination of several omics approaches to so-called trans-omics networks [123] can further facilitate unraveling occurring effects of xenobiotics [124]. Not only biomolecules of one type, e.g., proteins in proteomics, are identified with multi-omics approaches, but a systematic understanding of AOPs in response to xenobiotic exposure can be obtained [60]. Multi-omics approaches can combine different omics levels, thus resulting in comprehensive information on specific signaling pathways, which usually cannot be fully covered by one omics layer. Importantly, multi-omics approaches have already been considered for the investigation of immune metabolism and associated immunological dysfunctions [125]. Also, studying immunodeficiency has been discussed using multi-omics [126], and in medical research, multi-omics are considered to discover new therapeutics for chronic diseases or cancer [127,128,129]. Furthermore, the combination of several omics layers might improve our knowledge about the immune system [125,129,130,131,132].

To ascertain possible advantages of combining several omics layers for the examination of toxicological effects of xenobiotics in macrophages, we systematically reviewed publications that applied at least two omics approaches to xenobiotic-treated macrophages. We summarized the observed effects on pathway level and single analyte level, thus allowing conclusions about the optimal combination of omics layers.

## 2. Methods

**Figure 1:**
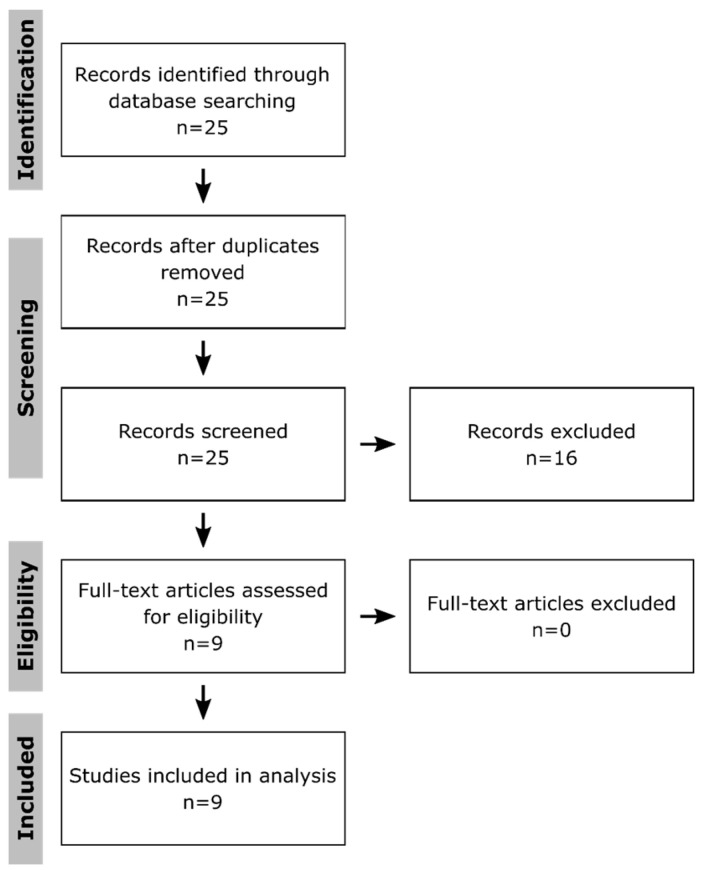
Selection of publications. Shown is the flow diagram according to the PRISMA guidelines [133] for systematic reviews. The publications were selected from the PubMed database, using the terms: “multi omics macrophages toxicity”, “multi omics macrophages toxicology”, “(proteomics AND transcriptomics AND metabolomics) macrophages toxicity”, “(proteomics AND transcriptomics AND metabolomics) macrophages toxicology”, “(proteomics AND transcriptomics) macrophages toxicity”, “(proteomics AND transcriptomics) macrophages toxicology”, “(proteomics AND metabolomics) macrophages toxicity”, “(proteomics AND metabolomics) macrophages toxicology”, “(transcriptomics AND metabolomics) macrophages toxicity”, and “(transcriptomics AND metabolomics) macrophages toxicology” on the 14 July 2020. Only publications describing original research results obtained from at least two omics approaches applied to xenobiotic-treated macrophages were further investigated. Furthermore, only publications using the English language were considered.

To systematically identify publications that applied multi-omics approaches to get insight into toxicological effects in macrophages, PubMed was searched on the 14 July 2020 using following terms: “multi omics macrophages toxicity”, “multi omics macrophages toxicology”, “(proteomics AND transcriptomics AND metabolomics) macrophages toxicity”, “(proteomics AND transcriptomics AND metabolomics) macrophages toxicology”, “(proteomics AND transcriptomics) macrophages toxicity”, “(proteomics AND transcriptomics) macrophages toxicology”, “(proteomics AND metabolomics) macrophages toxicity”, “(proteomics AND metabolomics) macrophages toxicology”, “(transcriptomics AND metabolomics) macrophages toxicity”, and “(transcriptomics AND metabolomics) macrophages toxicology”. The obtained 25 publications (Figure 1) were filtered for those that applied at least two omics approaches to macrophages after treatment with xenobiotics. Only original research articles using the English language were taken into consideration, while reviews and guidelines were excluded. Thus, nine publications were obtained, which were first compared regarding the used methods, numbers of replicates, investigated time points, the method to determine significant changes, the conduction of enrichment analyses, and the integration of the results to assess possible risks of bias at the study level. Subsequently, they were screened for affected pathways and key molecules. Thereby, it has to be kept in mind that the authors might have described a selection of effects in their publications.

## 3. Results

### 3.1. Summary of the Selected Publications

Based on the PubMed search, 25 publications were identified, of which nine publications contained original research results of xenobiotic effects in macrophages, applying two omics layers (Table 1). Interestingly, all nine publications used proteomics as one layer and either transcriptomics or metabolomics as the second one. No combination of transcriptomics and metabolomics was found. The fact that five publications considered a combination of proteome and transcriptome and four the combination of proteome and metabolome shows that there is no preferred approach so far.

To evaluate the quality of these nine publications, the applied method, the number of replicates, the time points, the method to determine significant changes, the conduction of enrichment analyses, and the integration of the results were assessed (Table 1). Most of the studies used p-values corrected for multiple testing, thus minimizing false-positive results. Furthermore, only in one publication, no pathway enrichment results were shown. Importantly, only one study integrated proteomics and metabolomics results before performing pathway enrichments. Several of the here screened studies used THP-1 cells as a macrophage model as performed for toxicological studies before [47]. Interestingly, five of the nine reviewed studies investigated the effects of NMs, thus highlighting that the use of multi-omics approaches has been already identified to be useful for nanotoxicology but also biasing the here described results towards NM toxicity.

### 3.2. Combination of Proteome and Transcriptome

Of the five publications comparing proteomics and transcriptomics results, four were investigating the effects of nanomaterials (NMs), indicating this combination to be already appreciated to assess the toxicological effects of NMs. Described affected pathways and key molecules of all five publications are summarized in the next section, starting with the four studies investigating NM effects.

Tilton et al., 2014 [134] determined the effects of TiO_2_ nanobelts and multi-walled carbon nanotubes (MWCNTs) in THP-1 cells, taking into account early and late time points. While the late time point was chosen to be after 24 h of exposure in both approaches, 1 h and 3 h were selected as early time points in transcriptomics and proteomics, respectively. Furthermore, enrichment analyses were conducted with both data sets, resulting in several enriched processes and the observation that most of the processes that were significantly enriched in the proteomic data set were also significantly enriched at the transcriptional level. To several of these processes, affected genes and proteins were assigned by the authors. Examples for relevant processes and assigned analytes were inflammation and apoptosis (NFκB, TNF, IL-8, MAPK, or PI3K), anti-apoptosis (BIRC5), cell cycle arrest, proliferation (MYC and CDK1), cytoskeletal functions, chemotaxis, and DNA repair (CHEK1, RAD51, and TOP2A). Furthermore, they highlighted that several regulated genes are known to be associated with the transcription factors aryl hydrocarbon receptor (AhR) and specificity protein 1 (Sp1), with AhR target genes like CYP1A1, CYP1B1, and ARNT2.

The effects of cationic gold NMs were investigated in THP-1 cells by Gallud et al., 2019 [135]. The authors performed transcriptomics after 6 h of sub-cytotoxic doses to capture early events and proteomics after 24 h and at a dose that triggered 50% cell death to elucidate perturbations linked to cell death. Thus, they found mitochondrial dysfunction to be significantly affected, leading to cell death with features of apoptosis and necrosis as well as inhibition of autophagy. Furthermore, pathways like oxidative phosphorylation, protein ubiquitination, and inflammation were significantly enriched. Mentioned dysregulated proteins and genes, belonging to these pathways were CYTC, NDUFA3, SOD2, AIF, mTOR, PHB, HMGB1, and PARK7. In summary, a high concordance of proteomics and transcriptomics results was observed.

Doumandji et al., 2020 [136] studied the effects of ZnO and ZnFe_2_O_4_ NMs in alveolar macrophages (NR8383) using 4 h exposure for transcriptomics and 24 h for proteomics. Importantly, proteomics was not only applied to cell extracts but also to cell culture supernatants. Besides the metal exposure responses that were observable in the transcriptomics data, they identified EIF2 signaling and thus protein homeostasis to be affected upon exposure as well as mTOR signaling, PDGF signaling, integrin signaling, and cholesterol biosynthesis. Furthermore, they described PLA2G16, S100A4, RPS27, MRPS15, TP53INP, SLFN3, AKAP9, ROCK1, SMARCA5, SMARCAD1, MED13, EEF1A2, ATF3, CYP4A8, RPL1, SDPR, MKI67, SMARCA5, HIPK2, CD68, CCL22, GDF15, and ALAS1 to be affected by the NMs. With proteomics, not only EIF2 signaling pathway and cholesterol biosynthesis, but also stress response, mitochondrial dysfunction, and sirtuin signaling were found to be enriched with changes in levels of IL1RAP, GPX4, LSS, PUM1, RPA2, TUBG1, and MAP1LC3B.

The effects of MWCNT in alveolar macrophages (NR8383) were examined after 4 h and 24 h exposure using transcriptomics and proteomics, respectively, by Nahle et al., 2020 [137]. Thereby, intracellular and secreted proteins were screened. Notably, the proteomics data were used to validate the results that were obtained using transcriptomics. Thus, they identified MWCNT-affected pathways and assigned genes and proteins. Among the list of pathways, translational processes with ribosomal proteins (RPS3, RPS12, RPL18, RPL39L, RPS27 and RPS14, RPS6KA1, RPS6KA4, MRPS2, and MRPS6), proteasomal proteins (PSMA2, PSMD8, and PSMD5), and translational proteins (EEF1G, EIF1B, EIF6, EIF2S2, EIF1A, EIF4EBP1, EIF1, EIF3M, EIF3K, TPT1, and EIF3) were described as well as cytoskeletal rearrangements based on tubulins (TUBA1A, TUBA4A, TUBB6, and TUBB2B), tubulin-building proteins (TUBB4B, TUBB5A, and TUB1AB) and chaperonins (CCT4, CCT8, CCT3, CCT2, CCT3, CCT4, and CCT8). Importantly, inflammatory responses (TLR2, CXCL2, CCRL2, IL6R, IL10RA, IL17RE, ILF2, CCL2, CCL4, IRAK2, IL7R, IL1B, IL1A, SOCS3, IFIT3, ANXA1, APOA2, C4A, CHIA, GPX4, KNG2L1, MAP2K3, PRDX2, STAT5B, VNN1, A2M, AGT, AHSG, MIF, THBS1, CKLF, CRLF2, IL10RA, IL17RE, IL3RA, NFIL3, and IFNGR2) were suggested to be a key event of MWCNT-induced toxicity. These responses were accompanied by autophagy (TFEB, ATG9A, ATG2A, ATG16L1, ATG101, and ATG4D), mTOR signaling (LAMTOR4, GRB2, IGF2BP2, VEGFB, GFER, and PDGFA), and inflammasome activation (NLRP1, NLRP3, NLRC4, AIM2). Furthermore, oxidative stress responses (RAS, HSPs, small MAF, PRDX1, MRP1, ABCC1, DNAJC7, MAFF, MAFG, and TXNRD1), and DNA damage responses (CHK1, CHK2, BRCA1, and RAD51) were described. Among the list of secreted proteins, VIM was most abundant. Again, transcriptomics and proteomics results were reported to be in accordance with each other.

Ihantola et al., 2020 [138] was the only of the here reviewed publications, combining transcriptomics and proteomics not to investigate the effects of NMs but of spruce and pine combustion emissions in murine macrophages (RAW264.7) after 4 h. They found glucocorticoid receptor signaling, cytokine signaling, endocytosis, NRF2-mediated oxidative stress pathways, unfolded protein response, cell cycle G2/M DNA damage checkpoint regulation, aryl hydrocarbon receptor signaling, and PPAR signaling to be enriched based on the transcriptomics data. In the proteome, oxidation–reduction processes, oxidative stress responses and endocytosis were shown to be affected as well as regulation of RNA- and transport-related processed. Importantly, several pathways, e.g., oxidative stress, cellular stress, and inflammation, were regulated at the transcriptome and proteome level, highlighting the consistency of the two omics approaches. Furthermore, in this study, the stress-inducible heat shock proteins HSPA1A and HSPA1B were described to be affected as well as further candidates like NFYC, SLC19A2, CGRRF1, MDM2, TSC22D1, and SYNE1.

### 3.3. Combination of Proteome and Metabolome

While five of the reviewed publications combined transcriptomics and proteomics, four combined metabolomics and proteomics. Latter ones are summarized in the following section.

Sapcariu et al., 2016 [122] investigated the effects of ship engine aerosol emissions on murine macrophages (RAW264.7). Based on changes in the metabolome, they found pro-inflammatory effects, indicated by changes in succinic acid, lactic acid, and itaconic acid levels as well as changes in DNA and RNA production based on adenine and uracil levels. Furthermore, an alternative metabolic phenotype was observed with changes in the TCA metabolites fumaric acid and glutamic acid. Effects to the immune response were confirmed by the proteome, where additionally endocytosis was found to be enriched. Especially, NFkB signaling was affected, as evidenced by changed TLR2, TNFAIP8L2, and PRDX2 levels.

The effects of the contact allergen 2,4-dinitrochlorobenzene (DNCB) were examined by Mussotter et al., 2018 in THP-1 cells [139]. Therein, alterations in the metabolome, and especially in phosphatidylcholines were observed, which were confirmed by changes of FAS within the proteome, suggesting metabolomic reprogramming of the THP-1 cells. Besides, spermine and taurine were found to be affected by the contact allergen. Furthermore, since acylcarnitines were found to be decreased in this study, diminished oxidative phosphorylation was assumed. Also, amino acid levels, as in the case of glutamic acid, as well as amounts of spermidine, changed upon treatment with DNCB. Based on the proteome, translation and unfolded protein responses with RLA2, HRNH1, PDIA1, and ENPL as candidates were described to be affected as well as mitochondrial activity and glycolysis with MDHM and KPYM, respectively.

In Marentette et al., 2019 [140], the effects of ethanol were explored by comparing liver infiltrating pro-inflammatory and anti-inflammatory macrophages. Enrichment analyses with significantly altered metabolites indicated effects on glycerophospholipid metabolism, arachidonic acid metabolism, and phospholipid biosynthesis. In contrast, antigen presentation, actin polymerization and organization, phagocytosis, and apoptotic regulation were found to be enriched based on the proteome. Furthermore, increases in phosphatidylcholines and linoleic acid were observed in this study, which were described to promote anti-inflammatory effects before [142,143].

Bannuscher et al., 2019 [141] applied proteomics and metabolomics to alveolar macrophages (NR8383) to get insights into NM mechanisms. Importantly, this was the only here reviewed publication that used proteins and metabolites for integrated enrichment analyses. Translational processes, glycolysis, chemokine signaling, oxidative stress responses, mitochondrial dysfunction, DNA damage response and cell death were among the described pathways. Furthermore, biomarker candidates were reported, e.g., PRDX2, GLO1, MRC1, THRAP3, SOD2, TRAP1, COX5A, EEFE1, and B2M as well as phosphatidylcholines, spermidine, putrescine, and amino acids like asparagine, histidine, and proline.

**Figure 2:**
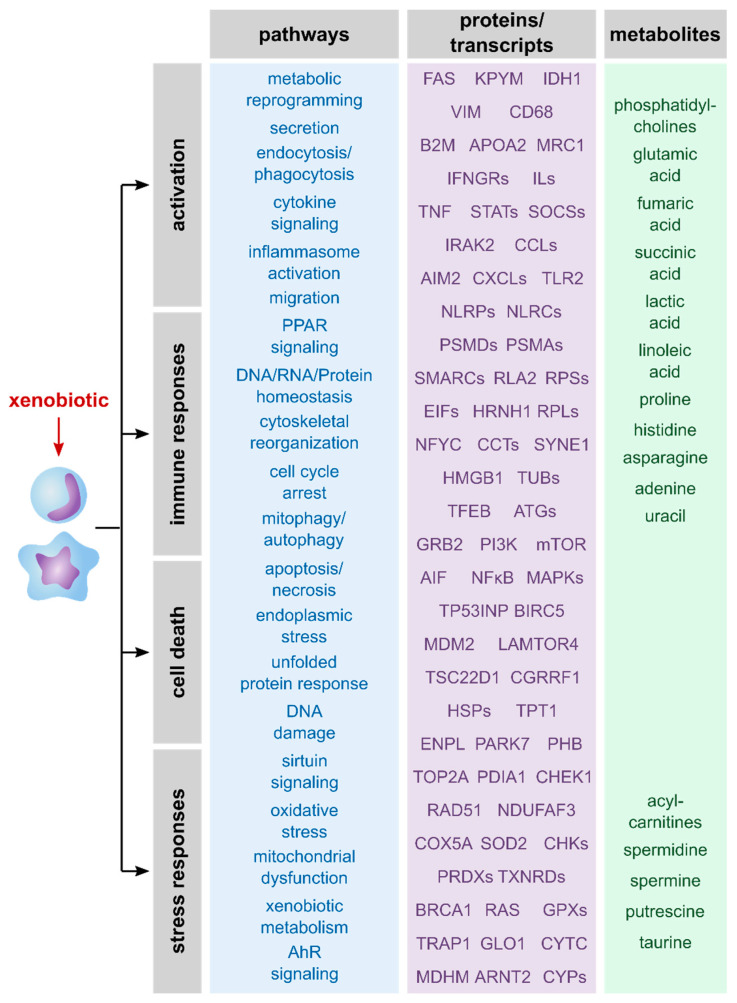
Overview of described effects and related transcripts, proteins, and metabolites. Shown are effects of xenobiotics on macrophages that were described in the here reviewed publications. Furthermore, examples of analytes are shown that give information about these processes. Notably, the analytes were roughly allocated to the related pathways and are assigned to the omics layer that can be used to identify and quantify them.

The summary of described pathways as well as proteins, genes and metabolites that were mentioned in the reviewed publications (Figure 2) reveals a broad spectrum of affected processes upon exposure of macrophages to xenobiotics. Taken together, macrophages typically respond to xenobiotic exposure by activation and immune responses, stress responses and/or cell death. Thus, all three omics layers allowed detailed information on xenobiotic-induced effects and should be considered relevant to unravel MoAs of xenobiotics.

## 4. Discussion

The summary of pathways and single molecules found to be affected in macrophages due to treatment with xenobiotics highlighted that valuable information on the MoAs of xenobiotics could be achieved from each of the here described omics layers. Thus, transcriptomics, proteomics, and metabolomics were shown to be suitable tools to unravel the MoAs of xenobiotics. Still, the combination of several omics layers led to a more comprehensive overview. Thus, with the application of multi-omics approaches, the definition of AOPs can be facilitated, hence highlighting also key molecules, which might be valuable biomarker candidates.

Comparing the information obtained from transcriptome, proteome, or metabolome, it is well-known that the transcriptome gives insights on the regulatory level and the metabolome about the phenotype with the environment directly influencing it. The proteome combines both layers, being a mediator between environmental conditions and cellular processes [144,145]. While for the combination of transcriptomic and proteomic data high accordance of the detected biological processes has been described [134,135,136,137,138], combining the proteome with the metabolome, also referred to as proteometabolomics [146,147,148,149,150], has shown the advantage of complementing each other, thus allowing for a better understanding of triggered processes upon macrophage treatment with xenobiotics. Thus, based on the here presented publications, the combination of proteome and metabolome seems to be preferable even though both combinations might be generally beneficial to decipher xenobiotics MoAs. Importantly, accompanying metabolomics data by proteomics or transcriptomics data has been suggested before to provide a comprehensive data basis for toxicological questions [151]. Unfortunately, none of the here reviewed studies used the combination of metabolome and transcriptome. When deciding whether to apply transcriptomics or proteomics, it has to be kept in mind that both may result in similar information on the overall pathway responses. However, there are also some differences. Transcriptomics allow the identification of more candidates, thus leading to a possibly more comprehensive data set. In contrast, the proteome is closer to the phenotype and combines several regulatory processes (e.g., protein expression, post-translational modifications, and protein–protein interactions) that lead to changes within the proteome but not the transcriptome [60,152].

Considering the number of available studies investigating one omics layer, it has to be noted that besides the integration of several omics layers within one study, as described here, also conducting integrational meta-analyses of data sets revealed in individual studies is possible. Thereby, it has to be ensured that the experimental design of the data that are going to be integrated match, to avoid misleading results, which is challenging since it depends on the completeness of metadata. Furthermore, multi-omics meta-analyses are hampered by the circumstance that not one repository is used for all omics approaches but different ones, e.g., the Proteomics Identification Database (PRIDE) [153] for proteomics, the Gene Expression Omnibus (GEO) [154] for transcriptomics, and MetaboLights [155] for metabolomics. Even for one omics layer, various repositories are available. However, recent efforts have been made to facilitate the identification of data that are suitable for multi-omics meta-analyses as in the case of The Omics Discovery Index (OmicsDI) [156] and the Multi-Omics Data set Finder (MOD-finder) [157].

Generally, one crucial point when comparing different omics layers is the selection of appropriate time points since there are differences in the kinetics of transcriptome, proteome, and metabolome. Transcriptional regulatory events typically occur earlier than protein changes [134], which was taken into account in most of the here presented studies. Importantly, changes in the metabolome occur between seconds and weeks due to the high diversity of existing metabolites [60]. Thus, choosing the right time point depends on the biological question and the target metabolites. In the here reviewed studies combining proteome and metabolome, always the same time points were investigated, which has the advantage of facilitating the integration of data from different omics layers using bioinformatics [60].

When integrating multiple omics data sets, the general differences between omics layers have to be considered, e.g., the number of identified features and applied normalization procedure. Furthermore, it has to be kept in mind that the directions of outcomes might vary among omics layers, thus hampering correlation analyses. However, plenty of tools for multi-omics data integration are available utilizing different bioinformatic procedures like correlation analysis, network construction, and the determination of similarities among others [158].

Notably, almost all the here reviewed studies analyzed the data from the different omics layers separately and then compared the results. For this purpose, enrichment analyses were conducted to identify significantly affected pathways based on significantly altered analytes, which is the standard procedure for omics data. In our opinion, investigating the changes based on pathway level is generally beneficial compared to considering single transcripts/proteins/metabolites since not the same analytes might be affected in two data sets due to distinct dynamics or depth of analysis for instance. Notably, those can still be part of the same pathway as recognized before [134].

Only one of the here reviewed studies [141] performed enrichment analyses based on combined omics layers, even though the integration of the omics layers for enrichment analyses is beneficial to reveal comprehensive insights into toxicological effects. It has been described before that the enrichment analysis of combined transcriptomics and proteomics data leads to more significant enrichment compared to the single omics layer, thus showing that the advantages of stronger p-values outweigh the disadvantage of noise that might be provoked by the combination of multiple omics layers [60]. Likewise, others have described the advantages of integrative multi-omics analyses for toxicological questions before [159,160]. Since tools for an integrated pathway enrichment are emerging [161,162], this should be considered in future multi-omics studies.

When combining proteome and metabolome for pathway enrichment, one might argue that this leads to biased results towards central carbon metabolism, oxidative stress, and processes related to translation and transcription because the usually identified metabolites play key roles in these processes. Notably, it should be kept in mind that usually way fewer metabolites are identified, compared to proteins or even transcripts, due to the state-of-the-art techniques. Thus, this bias should be negligible, and the advantages of combining several omics layers overweigh this issue in our opinion. Generally, methods should be applied that cover as many pathways as possible and not only one particular pathway. For example, changes in the central carbon metabolism are reported very often since the central carbon metabolism responds to stimuli sensitively. Unfortunately, these changes are rather unspecific since they can be related to various processes such as aging [163], cancer [164], diseases like non-alcoholic steatohepatitis (NASH) [165], and macrophage activation [166] for instance. Abundance-based information on metabolites involved in the central carbon metabolism, as obtained by metabolomics, do not necessarily give information whether the pathway was upregulated or downregulated. For example, increased metabolite abundances may result either from an elevated metabolism or an accumulation due to the inhibition of individual steps. This issue can be overcome by the use of metabolic flux analyses [167] or the investigation of additional omics layers [168].

Taken together, the here reviewed studies demonstrated that multi-omics approaches are well-suited to investigate toxicological effects because they allow the detailed analysis of the MoAs of xenobiotics. Linking changes identified by omics approaches and pathway perturbations to the phenotype by mapping them to AOPs has been considered promising for a facilitated risk assessment during the ECETOC workshop as well [63]. Also, based on a study combining genome-wide DNA methylation, mRNA, and microRNA data, it was concluded that the implementation of approaches based on multi-omics screenings along with systems biology-based multi-variate data modelling supports the building of more accurate AOPs [169].

Even though it has been concluded before that the use of multi-omics approaches is advantageous to decipher xenobiotics MoAs, this study has shown that multi-omics analyses in toxicology are still rare, especially when using macrophages as a model system. Macrophages are an essential component of the innate immune system and present in almost all tissues of the body [170]. They have been used as model systems for the investigation of effects of various xenobiotics, e.g., NMs [55,171], fungicides [172], herbicides [173], insecticides [174], and plasticizers [175] based on single parameters like toxicological endpoints, real-time PCR, or Western blotting before. However, studies applying omics approaches to investigate toxicological effects in macrophages are only emerging, and multi-omics studies for this purpose are still scarce. The circumstance that the majority of the here reviewed publications investigated the effects of NMs points out that nanotoxicology, as a pretty new research field, takes advantage of state-of-the-art multi-omics approaches for the analysis of macrophages. But in our opinion, multi-omics of macrophages are not only beneficial to unravel NMs MoAs but MoAs of any environmental stressor. Due to the enhancement of omics approaches and strategies for multi-omics data integration over the last years, we believe that this needs to be changed in the future. Thereby, not only the integration of transcriptome, proteome, and metabolome but also of further omics layers, e.g., epigenome [176], methylome [177], acetylome [178], and phosphoproteome [179], should be considered. Post-translational modifications (PTMs) are highly relevant since they regulate protein function, activity and thus, cell signaling [180]. PTMs like phosphorylations appear at comparably early time points after a stimulus [60], as shown for the protein tyrosine kinase c-Src kinase, which was activated 5 min after exposure to an environmental pollutant [181]. Noteworthy, the temporal behavior can differ between phosphopeptides [182]. In the case of epigenomics [183] in general and DNA methylation [184] in particular, there is no one correct time point to be investigated since the modifications are regulated dynamically. Thus, for those approaches, the explored time points have to be chosen depending on the research question as well, keeping in mind that an integrative analysis is facilitated by the use of paired samples [60]. Besides multi-omics studies on bulk samples as described here, the recent advances in single-cell omics studies might facilitate multi-omics studies on the single-cell basis in future as well [185,186].

Due to the ability of multi-omics studies to identify MoAs of xenobiotics, which facilitates the definition of AOPs, also the discovery of biomarkers is achievable based on omics as suggested by others in the context of diseases [187,188,189] and xenobiotics [141,190,191]. Notably, with the integration of omics data, the best suitable biomarkers for the particular biological context can be unraveled, which might belong to any of the omics layers.

**Figure 3:**
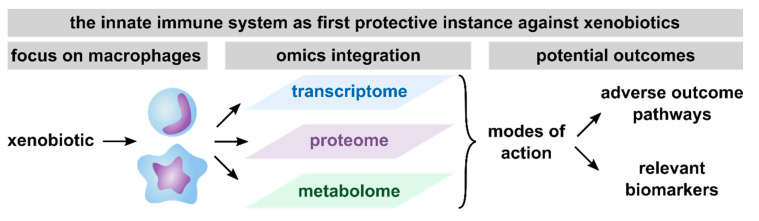
Summary of potential outcomes of multi-omics studies with a focus on macrophages. Presented are the outcomes that are achievable with the integration of transcriptome, proteome, and metabolome, including unraveling modes of action (MoAs), thus facilitating the definition of adverse outcome pathways (AOPs) and the identification of relevant biomarkers from any of the investigated omics layers.

In summary, based on the here reviewed studies, the conduction of multi-omics approaches has been proven useful to get detailed information on MoAs, thus facilitating the development of AOPs, the discovery of biomarker candidates and consequently, future risk assessment (Figure 3). Furthermore, macrophages were shown to be a relevant model system to assess the toxicity of xenobiotics, as indicated by the broad spectrum of pathways found to be affected in xenobiotic-exposed macrophages.

## 5. Conclusions

Here, we present a systematic review of multi-omics studies investigating the effects of xenobiotics in macrophages, leading to a broad spectrum of affected pathways and single transcripts, proteins, and metabolites. Thus, macrophages as a model system and multi-omics approaches as test strategy were proven suitable to unravel the MoAs of xenobiotics. As a consequence, multi-omics approaches should be applied more regularly to toxicological questions because they may facilitate the development of AOPs and thus, future risk assessment. Using macrophages as a model system will reveal in-depth insights into inflammatory processes, which are central KEs linking several AOPs.

## Figures and Tables

**Table 1:** Summary of the reviewed publications. Summarized are the used cell types (MΦ: Macrophages), the investigated xenobiotics, the applied omics approaches, the used method, the number of replicates, the investigated time points, the method to correct for multiple testing (Significance, n.d.: not defined), if enrichment analyses were conducted, and whether these were performed integratively. NM—nanomaterial; DNCB—2,4-dinitrochlorobenzene; UT—untargeted; Biocrates—use of one of the kits available from Biocrates; LFQ—label-free quantification; TMT—tandem mass tags; Diethyl—diethyl labeling; SILAC—stable isotope labeling by amino acids in cell culture; MA—microarrays; FDR—adjustment of p-values using false discovery rates; BH—*p*-value adjustment according to Benjamini & Hochberg; Dunnett—Dunnett’s T3 method to correct for multiple testing; ANOVA—analysis of variance; FC—fold change; SD—standard deviation.

Reference	Cell Type	Xenobiotic	Omics	Method	Replicates	Time Point	Significance	Enrichment	Integrative
Tilton (2014) [134]	THP-1	NM	Proteomics Transcriptomics	LFQ MA	5 3	3/24 h 1/24 h	FDR BH	Yes	No
Gallud (2019) [135]	THP-1	NM	Proteomics Transcriptomics	LFQ RNA-seq	3	24 h 6 h	FDR BH	Yes	No
Doumandji (2020) [136]	NR8383	NM	Proteomics Transcriptomics	LFQ MA	3 4	24 h 4 h	BH	Yes	No
Nahle (2020) [137]	NR8383	NM	Proteomics Transcriptomics	LFQ MA	4	24 h 4 h	ANOVA BH	Yes	No
Ihantola (2020) [138]	RAW264.7	Combustion emission	Proteomics Transcriptomics	Diethyl MA	3	4 h	Dunnett	Yes	No
Sapcariu (2016) [122]	RAW264.7	Ship engine emission	Proteomics Metabolomics	SILAC UT	4 4-5	4 h	n.d. ANOVA	Yes No	No
Mussotter (2018) [139]	THP-1	DNCB	Proteomics Metabolomics	SILAC Biocrates	3 5	4/8/24 h	FC and SD Bonferroni	No	No
Marentette (2019) [140]	MΦ	Ethanol	Proteomics Metabolomics	LFQ UT	3	4 weeks	ANOVA	Yes	No
Bannuscher (2019) [141]	NR8383	NM	Proteomics Metabolomics	TMT Biocrates	3-5 4-6	24 h	BH	Yes	Yes
